# Highly Efficient Micro-Scale Liquid-Liquid In-Flow Extraction of ^99m^Tc from Molybdenum

**DOI:** 10.3390/molecules26185699

**Published:** 2021-09-21

**Authors:** Petra Martini, Licia Uccelli, Adriano Duatti, Lorenza Marvelli, Juan Esposito, Alessandra Boschi

**Affiliations:** 1 Department of Translational Medicine, University of Ferrara, Via Fossato di Mortara, 70 c/o viale Eliporto, 44121 Ferrara, Italy; ccl@unife.it; 2 Department of Chemical, Pharmaceutical and Agricultural Sciences, University of Ferrara, Via L. Borsari, 46, 44121 Ferrara, Italy; dta@unife.it (A.D.); lorenza.marvelli@unife.it (L.M.); bsclsn@unife.it (A.B.); 3 Legnaro National Laboratories, National Institute of Nuclear Physics, Viale dell’Università, 2, 35020 Legnaro, Italy; juan.esposito@lnl.infn.it

**Keywords:** microfluidics, liquid–liquid extraction, technetium-99m, in-flow chemistry

## Abstract

The trend to achieve even more compact-sized systems is leading to the development of micro-scale reactors (lab-on-chip) in the field of radiochemical separation and radiopharmaceutical production. Technetium-99m extraction from both high and low specific activity molybdenum could be simply performed by MEK-driven solvent extraction if it were not for unpractical automation. The aim of this work is to develop a solvent extraction and separation process of technetium from molybdenum in a micro-scale in-flow chemistry regime with the aid of a capillary loop and a membrane-based separator, respectively. The developed system is able to extract and separate quantitatively and selectively (91.0 ± 1.8% decay corrected) the [^99m^Tc]TcO_4_Na in about 20 min, by using a ZAIPUT separator device. In conclusion, we demonstrated for the first time in our knowledge the high efficiency of a MEK-based solvent extraction process of ^99m^Tc from a molybdenum-based liquid phased in an in-flow micro-scale regime.

## 1. Introduction

Liver, kidney, brain, thyroid scans, imaging of bone lesions, and localization of myocardial infarctions are just some of the basic diagnostic tests performed daily with technetium-99m (^99m^Tc), a gamma ray emitting radioisotope (Eγ = 140 keV, t_1/2_ = 6 h) which covers over 85% of diagnostic applications in Nuclear Medicine. Technetium-99m is an everlasting radionuclide which has seen the birth of Nuclear Medicine, and the recent advances in technetium chemistry and detector technologies make it still modern and competitive with trendy PET radionuclides [1]. In the late 2000s, a recurrent lack of availability of ^99m^Tc in the hospitals due to a global shortage of molybdenum-99 (^99^Mo) because of frequent shutdowns of the ageing reactor-based ^235^U fission production chain, revealed the fragility of the traditional supply chain and, consequently, prompted the research community to look for alternative production routes.

Strong commitment has been devoted to the cyclotron-based direct production of ^99m^Tc, through the ^100^Mo(p, 2n)^99m^Tc nuclear reaction, as a valuable alternative [2,3,4,5,6,7,8,9,10,11,12]. Accurate studies on the cross section of this reaction and of collateral nuclear reactions have determined the optimal energy range (15–24 MeV) to maximize the production of ^99m^Tc, ensuring a radionuclidic purity level suitable for clinical applications [13]. This energy range is covered by most of the conventional medical cyclotrons already in operation in many hospital radiopharmacies, which can therefore be used for the production of ^99m^Tc on site ensuring a constant and on demand supply, without the aid of nuclear reactors.

Other reactor-based routes have been investigated based on the indirect production of ^99m^Tc induced by thermal or fast neutron beams, respectively through the ^98^Mo(n,γ)^99^Mo and ^100^Mo(n,2n)^99^Mo nuclear reactions, or gamma-ray beam ^100^Mo(γ,n)^99^Mo [11,14,15]. These routes are all affected by low specific activity molybdenum, being products and targets made of the same element material, thus complicating the generator-like extraction and separation process required for the isolation of high purity ^99m^Tc, since it would require large columns to adsorb molybdenum, decreasing the radioactive concentration of ^99m^Tc obtained to unacceptably low levels [16,17,18].

The extraction and purification of ^99m^Tc from the irradiated metal target is a key step in the production cycle, necessary to make the radioisotope suitable for further radiopharmaceutical processing and patient injection. This process must be simple, reproducible, and efficient, in order to rapidly supply a radioisotope with high purity. The automation of this process is essential to ensure these requirements and to minimize operators’ radiation exposure.

In this regard and in the framework of the LARAMED project of the Legnaro National Laboratories of the INFN, we developed an automatic module for the extraction, separation, and purification of cyclotron-produced ^99m^Tc from the molybdenum metal target, based on the solvent extraction technique [8,9,19]. This system exploits a helium-bubbling into a separation column to boost solvent extraction of technetium into a biphasic system, composed of an alkaline aqueous solution (containing Tc, Mo and contaminants) and an organic phase of methyl ethyl ketone (MEK). The developed system, although very efficient, may however be improved in terms of processing times and costs. Indeed, both dissolution and extraction-separation are the time-consuming steps of all procedures (about 20 and 30 min respectively, over 60 min total). Moreover, the developed module consists of an assembly of commercially-available modular units which are overall quite expensive (i.e., in the range 50–80 k€).

**Figure 1:**
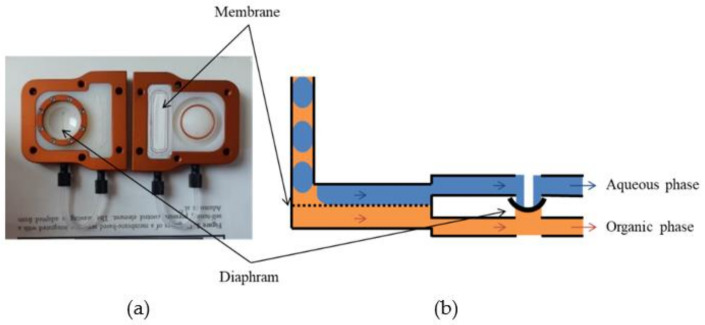
Picture (**a**) and scheme (**b**) of the ZAIPUT separation device.

The current trend in technology aiming to achieve even more compact systems is leading to the development of micro-scale reactors (lab-on-chip) in the field of radiochemical separation and radiopharmaceutical production, in order to improve performance and minimize chemical and radiological risks [20,21,22,23,24,25,26,27,28,29,30,31]. In this view, a latest generation device, the membrane-based Liquid–Liquid separator, 10 September (Figure 1), patented and produced by ZAIPUT Flow Technologies company (Cambridge, MA, USA), has been recently used for the radiochemical separation of radioisotopes of nuclear medical interest [23,29,30,32], for the miniaturization of liquid–liquid extraction processes in an in-flow chemistry regime. This device allows two immiscible phases to be effectively separated by exploiting interfacial tension and the affinity of one of the two phases for a microporous hydrophilic or hydrophobic polytetrafluoroethylene membrane (PTFE). Finally, thanks to a self-regulating differential pressure applied inside the device by a diaphragm, the separation can take place continuously [32].

The in-flow extraction process carried out at the micro-scale level creates some advantages when compared to similar processes performed on a macro-scale, including: shorter extraction times, an increase in the mass transfer coefficient, a better surface-volume interface ratio (S/V), etc. [31,33,34,35,36].

The aim of this work is to test the efficiency of the solvent extraction and separation process of technetium from molybdenum in an in-flow chemistry regime with the aid of the Zaiput separator. A versatile separation system that would allow for minimization of costs, times, dimensions, and volumes involved in the process, as well as being applicable also to ^99m^Tc indirect production methods, such as from low specific activity reactor-produced ^99^Mo, where the solvent extraction remains the best extraction-separation method [16,37].

## 2. Results and Discussion

In the production of radioisotopes for nuclear medicine, automation of the radiochemical separation process of the radioisotope of interest from the target and contaminants is necessary to make the results reproducible, minimize losses of radioactive material, decrease process times, and maximize recovery yields. Not least, automation allows for drastic reduction of radiation exposure to the operator conducting the separation.

Recently, we developed a separation and purification module based on the liquid–liquid extraction method applied to the cyclotron-production of ^99m^Tc from target of metallic molybdenum. Despite this system being extremely efficient, it requires rather long process times for the extraction of technetium with MEK with gas bubbling (helium or argon) within a glass separation column, and subsequent separation of the phases.

The purpose of this work was to develop a different, more compact, and efficient, automatic system for the extraction of ^99m^Tc from molybdenum, based on the liquid–liquid in-flow extraction process and separation with a membrane separator device, which would allow for minimization of costs, times, dimensions and involved volumes, while keeping high yield and quality.

The Zaiput device (Figure 1), a membrane separator, was therefore selected as the heart of the automatic system that allows two immiscible phases to be separated, taking advantage of the interfacial tension between them and the affinity of one of the two phases for a microporous membrane. Thanks to a differential pressure applied inside the device from a simple diaphragm, the separation can take place continuously [32]. This system allows the macro process of solvent extraction to be reduced to a micro scale, and therefore follows flow chemistry laws.

While the device takes care of the phase’s separation, the liquid–liquid extraction, in which the two phases are put in intimate contact allowing for solute transfer, takes place inside a capillary according to flow chemistry [38,39,40,41]. The capillary, optimized in terms of length and diameter and wrapped in a loop to minimize space, allows the two co-injected phases to alternate, forming a sort of train technically called the slug-flow regime. This typical micro-arrangement of phases is optimal for liquid–liquid extraction, as it allows the surface/volume ratio of the phases to be maximized, and therefore the ability to transfer solutes according to the chemical affinities involved [34,36,40].

**Figure 2:**
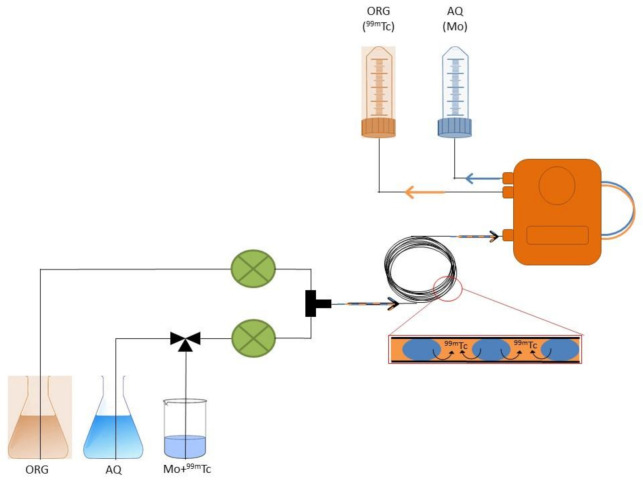
Diagram of the extraction and separation system.

In a preliminary phase, a prototype of the automatic system was assembled and tested with the aim of optimizing the flow rates of the biphasic system (NaOH 6 M/MEK) to achieve a proper separation. Subsequently, “cold” tests (without radioactivity) were performed to optimize the process conditions (volumes, transfer speed, slug-flow regime, loop dimensions, residence times of the sample in the loop, speed, and separation times). Capillary dimensions (ETFE capillary loop 1/8 inch, length = 106 cm; internal diameter 1.59 mm; internal volume about 2.1 mL) and flow rate (0.5 mL/min) are calibrated to maximize the contact between the two phases by achieving a slug-flow regime), the most suitable regime for liquid–liquid extraction on a micro-scale [40]. A schematic diagram of the overall system as it was assembled and installed is reported in Figure 2.

In order to assess how the efficiency of the extraction and separation system concerned, three “hot” tests (with radioactivity) were performed. The separation tests were carried out by adding ^99m^Tc-sodium pertechnetate eluted by a ^99^Mo/^99m^Tc generator in order to trace the activity in the process phases and to determine its efficiency. The collected results, summarized in Table 1, show that the system is able to quantitatively and selectively extract and separate (91.0 ± 1.8% decay corrected) the [^99m^Tc]TcO_4_Na in about 20 min.

After the phase separation, the ^99m^TcO_4_^−^ contained in the organic solution was purified through a silica and an alumina column, in order to remove molybdate traces and the MEK solvent, respectively. Finally, [^99m^Tc]TcO_4_^−^ was collected from the alumina column with saline. In Table 2, chemical (CP) and radiochemical (RCP) purity values of the [^99m^Tc]TcO_4_^−^ solution obtained at the end of the overall procedure is reported. Post-separation purification with column cartridges allows a pharmaceutical product compliant with the Pharmacopoeia standards to be obtained.

Given the high ^99m^Tc extraction yield and purity achieved, this system can be efficiently involved in the separation of technetium from both low and high specific activity molybdenum. In the case of indirect technetium production, the generator-like extraction of ^99m^Tc from low specific activity ^99^Mo can be performed running the protocol described here. Moreover, following further decay of ^99^Mo into ^99m^Tc, the molybdenum-rich aqueous phase coming out from the separator system, can be recirculated to the capillary loop and to the separator with fresh organic solvent for the extraction and separation of ^99m^Tc in continuous. Finally, the extraction process, starting with an alkaline aqueous solution of molybdenum, allows extension of the application of the system to not only the treatment of molybdenum metal targets, but also to oxides.

## 3. Material and Methods

### 3.1. Materials

The automatic system for the technetium/molybdenum liquid–liquid flow extraction on a microscale is composed of:

- a Zaiput SEP-10 device (purchased by ZAIPUT Flow Technologies company, Cambridge, MA, USA) mounting an OB-100 hydrophobic membrane, supplied with the device;
- two WPX1-U1/16S2-J8-CP peristaltic pumps (WELCO, Tokyo, Japan), flow rate of 0.5 mL/min at 6 V;
- two power supplies;
- a teflon T-junction;
- an ETFE capillary loop (1/8 “, length = 106 cm; internal diameter 1.59 mm; internal volume about 2.1 mL);
- ETFE tubes (1/8 “, internal diameter = 1.59 mm) and connectors.

Other materials involved in the process are: H_2_O_2_ 30% *w/w* (Titolchimica, Rovigo, Italy), methyl ethyl ketone (Carlo Erba, Milano, Italy), NaOH and sodium chloride 0.9% (Fresenius Kabi, Verona, Italy); [^99m^Tc]NaTcO_4_, in physiological solution eluted by a ^99^Mo/^99m^Tc generator (UTK, Curium, London). Silica and acidic alumina SepPak Cartridges were purchased from Waters Corporation (Milford, MA, USA). Natural molybdenum metal target (^nat^Mo, 99.95% purity, 150 mg) were produced by Spark Plasma Sintering technique (SPS) [46].

### 3.2. Procedure

A molybdenum metal target, not irradiated, was dissolved in hot concentrated H_2_O_2_ 2 mL, and subsequently 3.5 mL of NaOH 6 M was added to convert MoO_3_ into Na_2_MoO_4_.

In order to “mimic” the irradiated target conditions, a few microliters of generator eluted [^99m^Tc]-sodium pertechnetate were added to the target solution. Subsequently, the sample containing the mixture of Mo and Tc (about 5 mL), together with the organic phase MEK (5 mL), was pumped through the extraction-separation system, previously conditioned with the biphasic NaOH/MEK system (5 mL/phase).

**Figure 3:**
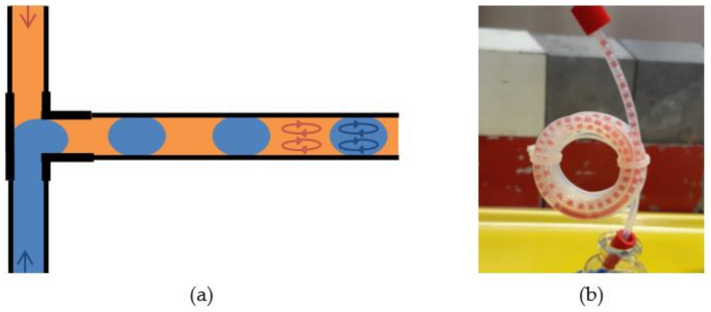
(**a**) Operating principle with schematic representation of internal circulation within the two phases and (**b**) photo of a slug-flow regime in the loop in which the organic phase has been colored red by adding a dye.

The two phases converge, by means of a Teflon T-junction, in the capillary loop by forming a kind of train technically called the “slug-flow” regime (Figure 3b). The residence time in the loop is approximately 2.5 min and, at this stage, the transfer of [^99m^Tc]TcO_4_^−^, from the aqueous to the organic phase occurs. Exiting from the loop, the two phases enter the ZAIPUT separator device, from which the technetium-rich organic and molybdenum-rich aqueous phases come out separated (ORG/AQ OUT 1 for the preconditioning, ORG/AQ OUT 2 for the sample).

Once drained of all the sample, a washing with the biphasic NaOH/MEK system (5 mL/phase) was performed to collect any residual sample in the system (ORG/AQ OUT 3). Between one experiment and another, the system was washed with water and MEK. All the samples collected at the output of the ZAIPUT device were then measured with an activity calibrator to detect the amount of ^99m^Tc activity present in each sample, thus determining the extraction yield. The technetium was then extracted and purified from the ^99m^Tc-rich organic phase with a silica and alumina column in series, preconditioned with 5 mL of MEK and 10 mL of deionized water, respectively. The technetium was then eluted from the alumina column with 3 mL of saline solution.

### 3.3. Quality Controls

Paper chromatography was carried out on Whatman 1 strips using methanol/water (8:2) and Physiological solution as mobile phases, Chromatograph Cyclone Plus Storage Phosphor System and Software Optiquant ™.

Kit Merckoquant Molibdeno 5–250 MG/L was purchased from Merck (Darmstadt, Germany) and Kit Tec-Control Aluminium Breakthru from Biodex (Shirley, NY, USA).

Dose Calibrator Capintec CRC-15R (Ramsey, NJ, USA) was used for activity measurements.

Gas-chromatography (GC) was performed using Agilent-Pal H6500-CTC injector, gas-chromatograph Agilent Gc 6850 Series II Network, mass spectrometry detector (MS) Agilent Mass Selective Detector 5973 Network and column Agilent J&W DB-624 UI (20 m, 0.18 mm, 1.00 u) was used for chromatographic separation.

The operating conditions of the headspace were: incubation time: 50 min; incubation temperature: 80 °C; magnetic stirring speed: 250 rpm. The column oven temperature was set at 35 °C and remained constant for 4 min. After this time, the temperature was raised up to 240 °C at 15 °C/min. A constant column flow of 0.7 mL/min of helium was used.

## 4. Conclusions

In this work we demonstrated for the first time the efficiency of the MEK-based solvent extraction process of ^99m^Tc from a molybdenum-based liquid phased in an in-flow micro-scale regime, using a compact ZAIPUT separator device. Further developments will be carried out aimed at completing automation of the system thereof, including the dissolution and post-separation purification phases.

This system allows the extraction time and separation of technetium from the organic phase to be drastically reduced and can be used both to purify technetium from molybdenum metal targets in the direct cyclotron ^99m^Tc production, as well as in indirect ^99m^Tc production such as the ^99^Mo production, by irradiating a natural molybdenum using a 14-MeV accelerator-driven neutron source.

## Figures and Tables

**Table 1:** Average percentage of extraction ^99m^Tc (*n* = 3). (ORG/AQ OUT 1: preconditioning, ORG/AQ OUT 2: sample collection, ORG/AQ OUT 3: residual washing).

Sample	Volume [mL]	^99m^Tc %
Sample vial	empty	3.3 ± 0.7
ORG OUT 1	4	-
AQ OUT 1	5	-
ORG OUT 2	8.5	91.0 ± 1.8
AQ OUT 2	7	2.1 ± 0.6
ORG OUT 3	2.5	2.2 ± 1.4
AQ OUT 3	3	1.5 ± 2.1
H_2_O washing	5	-
MEK washing	5	-

**Table 2:** CP and RCP of [^99m^Tc]TcO_4_^−^ obtained at the end of the overall procedure of this work compared with the European pharmacopeia requirements for injection, other solvent-extraction-based techniques from the literature, and the quality of [^99m^Tc]TcO_4_^−^ eluted from a UTK generator (Curium). * EU Phar. = European Pharmacopoeia.

Parameters		This Work	EU Phar. * [42,43,44,45]	Other SE-Based Techniques [16,37]	Generator Eluate
**CP**	pH	5	4–8	6–7	6
	Mo	<5 ppm		<10 ppm	-
	Al	<5 ppm	<5 ppm	<10 ppm	<5 ppm
	MEK	<0.0006% (*v/v*)	<0.5% (*v/v*)	<0.1% (*v/v*)	-
**RCP**	^99m^TcO_4_^−^	>98%	≥95%	>99%	>99%

## Data Availability

Not applicable.
